# Healthcare Workers’ Perspectives on 24-h Shift Work in Latvia: A Cross-Sectional Study

**DOI:** 10.3390/healthcare14182993

**Published:** 2026-09-13

**Authors:** Olga Cerela-Boltunova

**Affiliations:** Department of Nursing and Midwifery, Riga Stradiņš University, LV-1067 Riga, Latvia; cerela.olga@gmail.com or 039389@rsu.edu.lv; Tel.: +371-28178990

**Keywords:** 24-h shifts, healthcare workers, nurses, shift work, occupational fatigue, patient safety, work schedule, workforce retention, Latvia

## Abstract

**Highlights:**

**What are the main findings?**

- Healthcare workers working extended shifts reported substantial fatigue, emotional exhaustion, health complaints, and perceived patient-safety concerns, while a substantial proportion still preferred to retain 24-h shifts.
- Frequently reported reasons for retaining 24-h shifts included financial considerations, schedule convenience, consecutive days off, and the possibility of combining employment across multiple workplaces.

**What are the implications of the main findings?**

- Future work-schedule reforms should consider staffing capacity, remuneration, opportunities for recovery, schedule flexibility, and healthcare workers’ preferences.
- The findings support further evaluation of flexible and mixed scheduling approaches rather than immediate universal replacement of 24-h shifts.

**Abstract:**

**Background/Objectives:** Prolonged working shifts may adversely affect healthcare workers’ health, well-being, cognitive performance, and patient safety. Nevertheless, 24-h shifts remain commonly used in Latvian healthcare. This study aimed to examine healthcare workers’ experiences of 24-h shift work, their attitudes toward revising this work schedule, and the main reported reasons underlying their preferences. **Methods:** A cross-sectional survey was conducted in Latvia between October 2025 and February 2026. A structured 64-item questionnaire assessed sociodemographic and professional characteristics, workload, shift patterns, health and emotional well-being, perceived patient-safety concerns, professional motivation, and attitudes toward alternative work schedules. Of 1524 submitted questionnaires, 1318 were included in the analysis. Quantitative data were summarized using descriptive statistics, while open-ended responses were examined using qualitative content analysis. **Results:** Most respondents regularly worked extended shifts, and 40.5% worked at more than one institution. More than half reported health changes that they perceived as potentially related to extended shift work, with 61.1% reporting emotional exhaustion and 49.2% reporting persistent fatigue. Fatigue during extended shift work had contributed to a self-reported error or perceived decline in care quality among 34.9% of respondents. Despite these concerns, a substantial proportion of participants preferred to retain 24-h shifts. Frequently reported reasons included schedule convenience, consecutive days off, financial considerations, and the ability to combine employment across multiple workplaces. Open-ended responses further highlighted preferences for flexibility, employee choice, adequate staffing, opportunities for rest, and consideration of financial consequences when revising work schedules. **Conclusions:** The findings demonstrate a tension between healthcare workers’ perceived health and patient-safety concerns associated with extended shift work and the practical and financial advantages associated with retaining 24-h shifts.

## 1. Introduction

The organization of working time in healthcare is one of the most important factors affecting both the professional resilience and health of staff and patient safety and quality of care. In many countries, long working shifts, including 24-h schedules, have been associated with increased fatigue, insufficient recovery, and concerns regarding patient safety and quality of care [1]. In Latvia, 24-h shifts remain widespread, especially in emergency medicine, anaesthesiology, and intensive care, and this practice is driven by a shortage of resources, tradition, and practical considerations such as commuting options or combining work at several medical institutions [2,3]. These circumstances make the organization of working time an important issue for both healthcare workers and the sustainability of healthcare services.

Some Latvian studies consistently emphasize the link between long shifts and staff fatigue, sleep disturbances, and emotional exhaustion [4,5]. Research by Trinkoff and colleagues (2011) [6] confirmed that nurses working shifts longer than 12 h more often experience health problems and care errors. Similar findings are reported by Griffiths et al. (2014) [7], who emphasize that shift length directly correlates with threats to patient safety. Burnout has also been associated in previous research with excessive workload and insufficient recovery; however, burnout itself was not assessed using a validated instrument in the present study [8].

From the patient perspective, international evidence has demonstrated associations between staffing, working conditions, and patient outcomes [9,10]. These findings indicate that the organization of working time is not only an issue of staff well-being but may also be relevant to the quality and efficiency of healthcare delivery.

In Latvia, the question of revising 24-h shift work has also become relevant at the policy level [5], with proposals concerning a transition toward shorter or three-shift work models. This discussion takes place in the context of persistent staff shortages, high workloads, and the frequent need for healthcare professionals to combine employment across more than one institution. At the same time, healthcare workers may perceive practical benefits of 24-h schedules, including fewer commutes, more consecutive days off, and the possibility of combining several workplaces.

Therefore, it is important to understand not only the perceived disadvantages of 24-h shifts but also the reasons why healthcare workers may prefer to retain this work pattern.

For this reason, a study was conducted within Latvia in which 1524 respondents took part, of whom 1318 questionnaires were valid for analysis. The aim of this study was to examine healthcare workers’ experiences of 24-h shift work, their attitudes toward revising this work schedule, and the main reported reasons underlying their preferences. The results provide a substantial contribution to the discussion on work-schedule reforms in Latvian healthcare, while also serving as a basis for the further development of policy and practical solutions.

## 2. Materials and Methods

### 2.1. Description of the Survey

A cross-sectional survey was conducted in Latvia using a structured 64-item questionnaire developed specifically for this study and distributed via the Google Forms platform. The survey aimed to explore healthcare workers’ experiences and opinions regarding 24-h shift work, its perceived effects on health, professional well-being, patient safety, and work organization, as well as their attitudes toward potential revision of current 24-h shift practices in Latvia. A non-probability voluntary-response sampling approach was used. The survey was anonymous, took an average of 15–20 min to complete, and respondents could withdraw from participation at any time before submitting the questionnaire. The study was conducted between 3 October 2025 and 19 February 2026.

The target population consisted of healthcare professionals working in Latvia. The questionnaire was distributed through professional healthcare organizations and institutions, as well as through online communication channels and professional networks, to ensure broad accessibility across different healthcare professions and workplaces. The inclusion criterion was current employment as a healthcare professional in Latvia during the study period. Participation was voluntary, and no restrictions were applied regarding profession, clinical specialty, workplace, or region. A total of 1524 questionnaires were submitted, of which 1318 were included in the final analysis. Submissions that did not contain complete responses to the required structured questionnaire items were considered incomplete and excluded from the analysis (n = 206). The open-ended questions were optional and were not used as a criterion for questionnaire completeness or exclusion. The dataset was also checked for potential duplicate submissions before the final analysis.

The questionnaire was structured into several thematic sections. The first part covered sociodemographic data and professional profile—age, gender, education, position (nurse, nursing assistant, physician assistant, midwife, radiology assistant, senior nurse, etc.), length of service in healthcare, current workplace and department, as well as workload at a single institution and overall. The second section gathered information on work schedules and experience with 24-h shifts—the work regime (day work, shift/on-call work, or night work), the frequency and intensity of such shifts, opportunities to rest or sleep during work, and the compatibility of work schedules with family life and other personal needs. Questions concerning fatigue, health effects, recovery, and professional functioning were answered by respondents working extended shifts, including both 16- and 24-h shifts. For the purposes of these analyses, the term “extended shifts” refers to both 16- and 24-h shifts. Therefore, respondents who reported that they did not currently work 24-h shifts were not necessarily without experience of prolonged shift work, as some worked 16-h shifts. Percentages for the required structured questionnaire items were calculated using the 1318 questionnaires included in the final analysis as the denominator, unless otherwise specified. Open-ended questions were optional; therefore, the denominator for each qualitative analysis was the number of respondents who provided a response to that particular question. The third section was devoted to health and well-being aspects, in particular fatigue and exhaustion after extended shifts, physical complaints (for example, headaches and blood pressure fluctuations), and perceived effects on emotional well-being and stress, as well as sleep quality and recovery opportunities. No formal pilot testing or psychometric validation was performed.

The next section focused on patient safety issues—respondents’ perceptions of the relationship between fatigue and the risk of errors, as well as their perceived ability to make professional decisions after prolonged working hours. This was followed by questions on respondents’ attitudes toward revising 24-h shifts, both at their specific workplace and at the national level, including possible benefits and risks of moving to 8- or 12-h shifts, as well as preferences regarding different shift lengths (8, 12, 16, or 24 h). Information was also gathered on motivation to continue working in the profession and factors that reduce this motivation.

An important part of the questionnaire consisted of open-ended questions, in which respondents could freely express their opinion on the reasons why they support or oppose revising 24-h shift practices, as well as offer suggestions for improving work organization, scheduling, and fatigue reduction. This approach made it possible not only to quantitatively assess trends but also to gain qualitative insight into healthcare workers’ experiences and reasoning.

The questionnaire was developed specifically for this study based on relevant scientific literature, national policy documents, and consultation with healthcare professionals with practical expertise in work-schedule organization and 24-h shift practices, including professionals involved in relevant professional associations. Before data collection, the questionnaire items were reviewed by healthcare professionals with experience in this field to assess their relevance, clarity, and applicability to the Latvian healthcare context. The questionnaire was administered in Latvian, and the complete questionnaire, including all questions and response options, is provided in the Supplementary Materials.

The survey was designed to provide a broad view of healthcare staff experiences and perspectives across various departments and positions, while respecting participant anonymity and data security. Respondents’ answers were analysed and reported in aggregate form and used for research and healthcare work-organization improvement purposes.

No a priori sample-size calculation was performed; the study aimed to obtain the broadest possible voluntary participation of healthcare professionals during the predefined data-collection period. Because the survey link was distributed through multiple professional organizations, institutions, and open communication channels, the total number of healthcare professionals who received or viewed the invitation could not be determined; therefore, a response rate could not be calculated.

### 2.2. Statistical Analysis

Quantitative data were analysed using IBM SPSS Statistics, version 29.0 (IBM Corp., Armonk, NY, USA). Continuous variables, including respondents’ age and length of professional experience, were summarized using means, standard deviations, and ranges. Categorical and ordinal variables were presented as absolute frequencies and percentages.

Descriptive analyses were performed to characterize the study population, working patterns, experiences related to 24-h shifts, and respondents’ attitudes toward potential changes in work schedules. Percentages were calculated using the number of valid responses available for each respective questionnaire item as the denominator. Open-ended questions were optional; therefore, the denominator for each qualitative analysis was the number of respondents who provided a response to that particular question.

#### Qualitative Analysis

Responses to the open-ended questions were analysed using inductive qualitative content analysis. The unit of analysis was each respondent’s written response to the respective open-ended question. Responses were reviewed by four nurses, each with more than 20 years of professional experience in healthcare and more than 10 years of management experience. The open-ended responses were divided among the four reviewers for initial coding according to recurring meanings and concepts. The resulting codes and thematic categories were subsequently reviewed collectively. Differences in interpretation and ambiguous responses were discussed among the reviewers and resolved through consensus. Similar codes were grouped into broader categories, themes, and subthemes. Where a response contained more than one distinct idea, multiple codes could be assigned to the same response. No dedicated qualitative-analysis software was used. The resulting thematic categories were used to summarize the main patterns identified in respondents’ answers. As the open-ended questions were optional, exact frequencies were calculated using the number of respondents who answered each respective question as the denominator.

### 2.3. Ethical Consideration

The study was conducted in accordance with the Declaration of Helsinki [11] and relevant Latvian regulations [12,13]. Ethical approval was obtained from the Ethics Committee of Riga Stradiņš University (Decision No. 2-PĒK-4/416/2023, 9 May 2023). Participation was voluntary, and all data were anonymised. No patient-identifiable data was collected.

Participation of healthcare professionals was voluntary. Before beginning the online questionnaire, participants were provided with information regarding the purpose of the study, voluntary participation, anonymity, and data processing. Proceeding with and submitting the questionnaire after reading this information was considered confirmation of informed consent to participate in the study. No directly identifiable personal or patient data were collected.

## 3. Results

### 3.1. Characteristics of Respondents

The study included 1524 respondents, of whom 1318 questionnaires were valid for analysis. The average age of participants was 42.24 years (SD = 12.35; range from 19 to 73 years). The average length of service in healthcare reached 15.50 years (SD = 12.74; range from 0.3 to 52 years).

The gender distribution showed that the study sample consisted predominantly of women (95.1%, n = 1254), while men accounted for only 4.3% (n = 57), and 0.5% (n = 7) chose not to disclose their gender.

In terms of education, the largest share of respondents had obtained secondary vocational education (23.1%, n = 305) or second-level professional higher education/a bachelor’s degree (41.4%, n = 545). In addition, 21.7% (n = 286) had first-level professional higher education, 10.4% (n = 137) held a master’s degree, and only four respondents were doctoral-level graduates (0.3%).

More than half of the respondents were nurses (56.4%, n = 744). Nursing assistants also formed a relatively large group (11.8%, n = 156). Representation in other positions was much smaller—physician assistants (9.6%), medical assistants (4.7%), midwives (3.3%), senior nurses (3.6%), and others with a very small share.

The largest professional groups by clinical area were represented by surgical departments (n = 256), Emergency Medical Services (EMS) (n = 213), and intensive care units (ICU) (n = 202). Participants from several other clinical areas were also represented in the sample.

### 3.2. Workload and Experience with Shifts of Different Lengths

**Figure 1:**
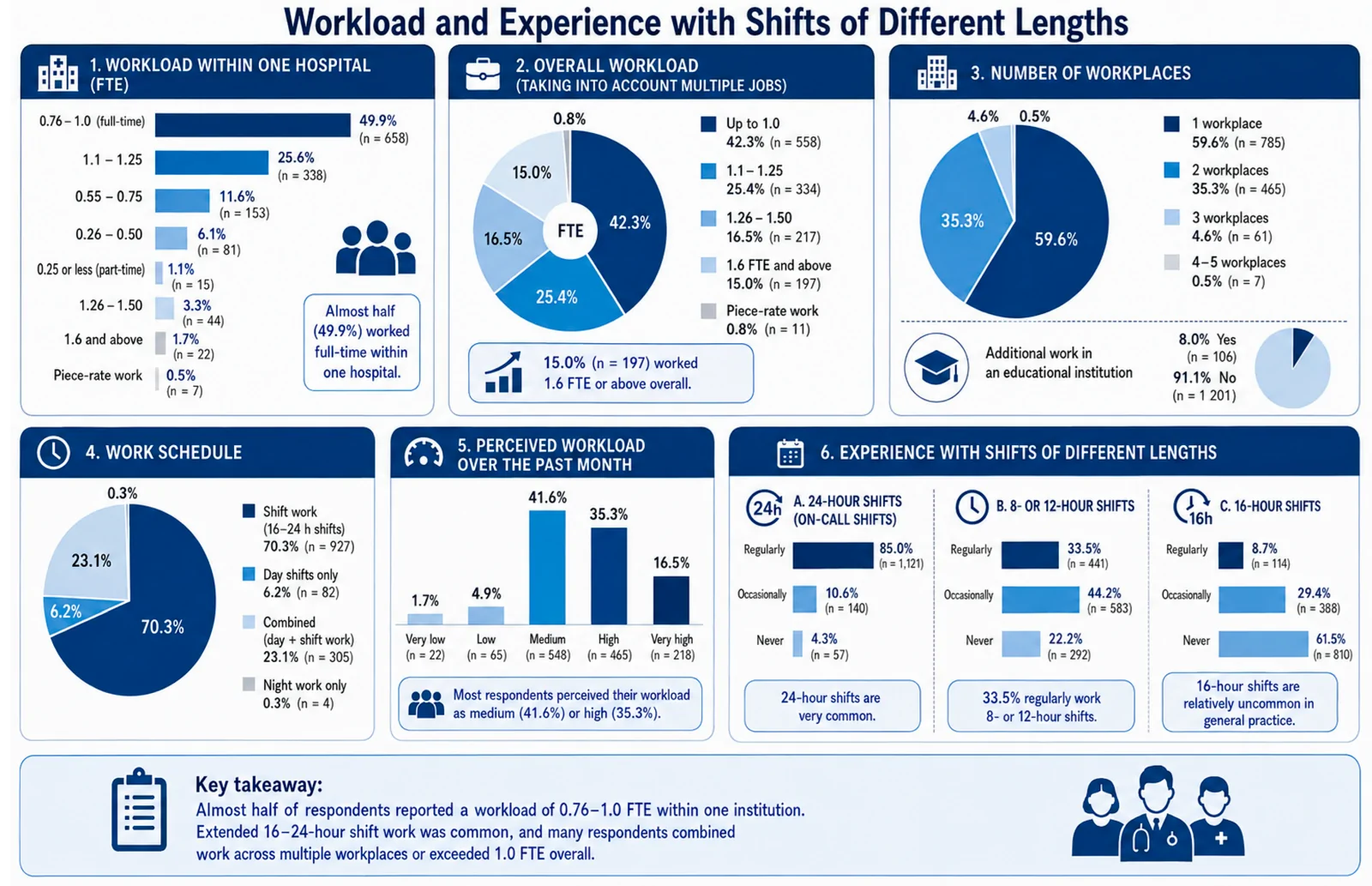
Workload and experience with shifts of different lengths.

Figure 1 summarizes respondents’ workload, number of workplaces, work schedules, perceived workload, and experience with shifts of different lengths. Almost half of respondents (49.9%, n = 658) reported a workload of 0.76–1.0 FTE within one institution, while 56.8% reported an overall workload exceeding 1.0 FTE when employment across multiple workplaces was considered. More than one-third (35.3%) worked at two workplaces. Shift work involving 16–24-h shifts was reported by 70.3% of respondents. Regarding 24-h shifts, 66.0% reported working such shifts several times per week and 19.0% approximately once per week. In contrast, 8.6% regularly worked 16-h shifts, while 33.5% regularly worked 8- or 12-h shifts. Most respondents rated their workload as medium (41.6%) or high (35.3%).

### 3.3. Perceived Impact on Health and Emotional Well-Being

More than half of respondents (54.9%, n = 724) reported having noticed changes in their health that they perceived as potentially related to extended shift work. In addition, 25.9% (n = 342) reported having consulted a doctor for health issues that they considered possibly related to extended shift work.

Emotional well-being ratings showed that 51.7% of respondents either rather or fully agreed that extended shifts negatively affected their emotional well-being, while 29.4% either rather or fully disagreed. More than half of respondents (61.1%, n = 805) reported experiencing emotional exhaustion during the previous six months, while 49.2% (n = 646) reported persistent fatigue.

Overall, 34.9% (n = 460) reported that fatigue during extended shift work had contributed to an error at work or a perceived decline in care quality, while 32.8% (n = 432) reported that this had never occurred and 32.3% (n = 426) were uncertain.

Regarding clinical decision-making, 12.0% reported a substantial perceived effect of fatigue on their ability to make safe and accurate clinical decisions, 23.4% reported a slight perceived effect, and 56.1% reported no substantial perceived effect. When asked specifically about working for more than 20 h, 37.4% reported sometimes feeling unable to make correct professional decisions, while 6.4% reported this often and 1.1% always.

### 3.4. Attitudes Toward 24-h Shifts and Their Future

**Figure 2:**
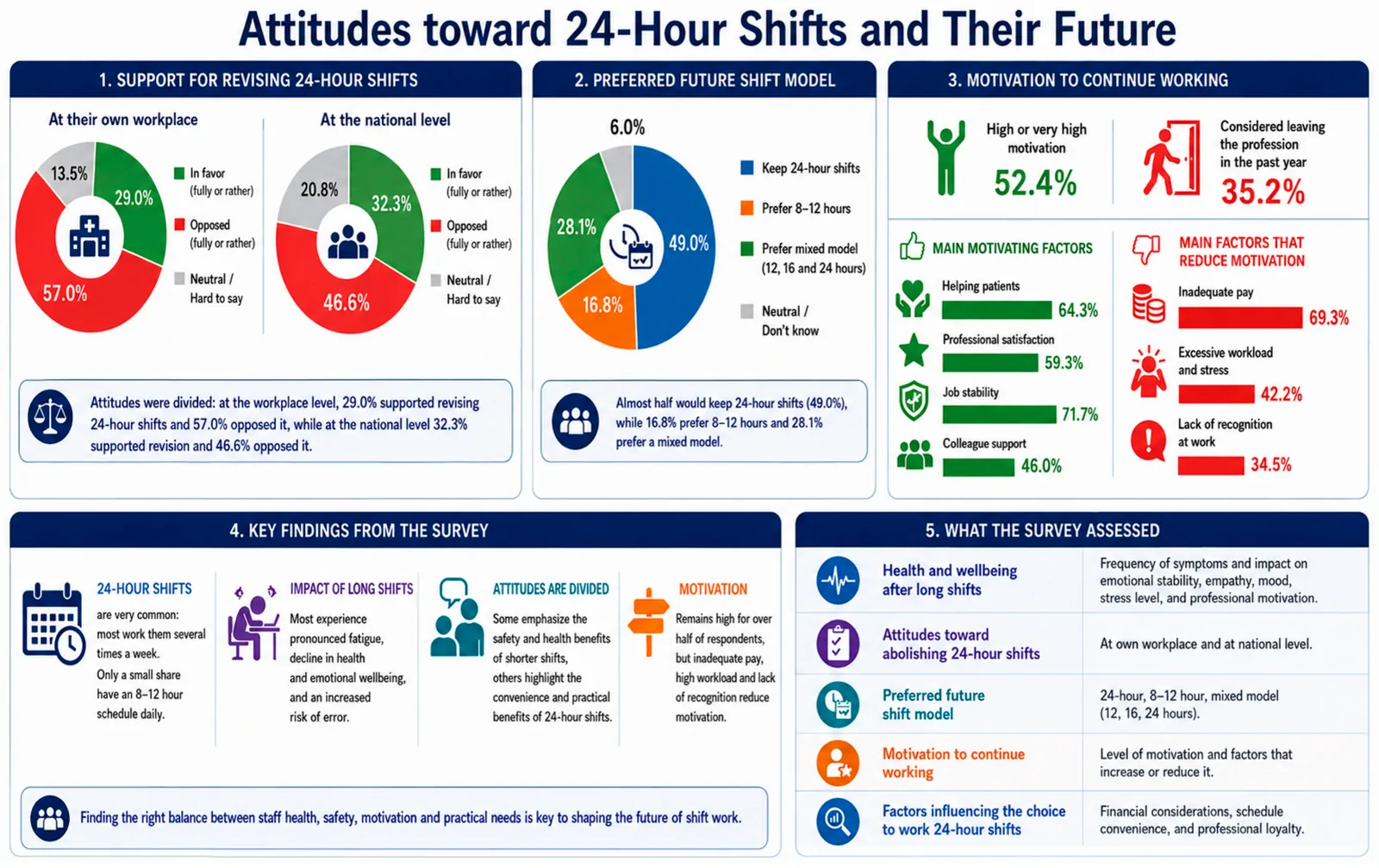
Attitudes toward 24-h shifts and their future.

Attitudes toward revising 24-h shifts were divided. At respondents’ own workplaces, 29.0% supported replacing 24-h shifts with shorter schedules, while 57.0% opposed such a change and 13.5% were neutral. At the national level, 32.3% supported revising 24-h shift work, 46.6% opposed it, and 20.8% were neutral or uncertain (Figure 2).

To further characterize respondents’ experiences of extended shift work, participants were asked to report the frequency of various symptoms following extended shifts and to rate the perceived effects of prolonged working hours on emotional stability, empathy toward patients, mood, stress level, and professional motivation. In addition, respondents rated the importance of several factors considered when choosing to work 24-h shifts, including financial considerations, schedule convenience, and professional loyalty. The results are summarized in Table 1, Table 2 and Table 3.

**Figure 3:**
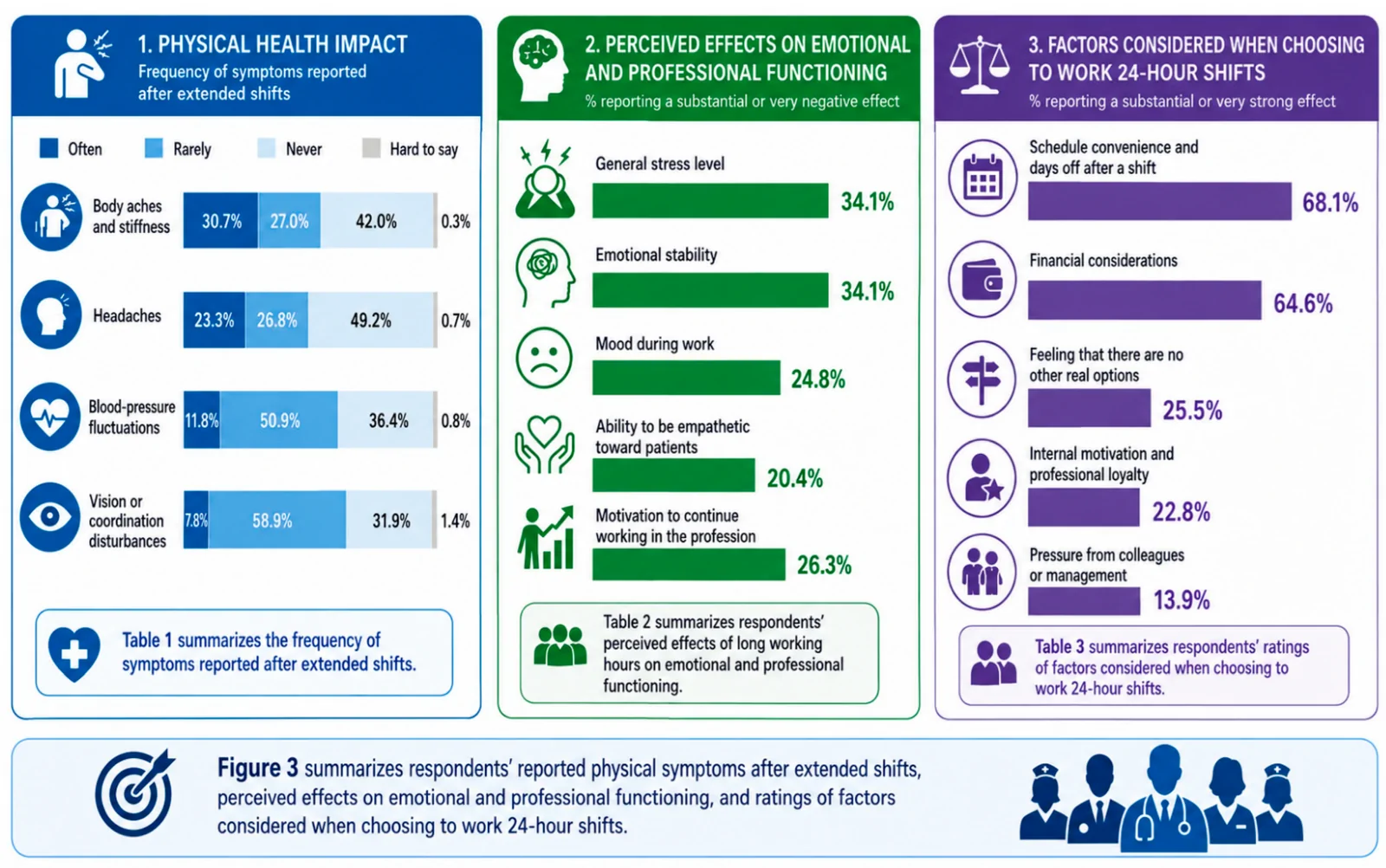
Perceived effects of extended shifts and factors considered when choosing 24-h shifts.

Table 1, Table 2 and Table 3 summarize respondents’ reported physical symptoms following extended shifts, perceived effects of prolonged working hours on emotional and professional functioning, and ratings of factors considered when choosing to work 24-h shifts. Among the factors assessed, schedule convenience and days off after a shift received the highest proportion of substantial or very strong ratings (68.1%), followed by financial considerations (64.6%). These findings are further summarized in Figure 3.

### 3.5. Open-Ended Questions

The survey also included six open-ended questions to obtain a more detailed picture of hospital staff experiences and perspectives regarding 24-h shift work. In these questions, respondents shared their arguments for or against retaining 24-h shifts, identified the main benefits and risks, described their personal experiences with fatigue, health problems, and perceived error risk, and offered suggestions for possible alternative approaches to work-schedule organization. Responses to these questions were analysed using inductive qualitative content analysis, as described in the Methods section. As the open-ended questions were optional, the number of responses varied across individual questions and is reported separately for each analysis.

#### 3.5.1. Health Problems Reported After 24-h Shifts

**Figure 4:**
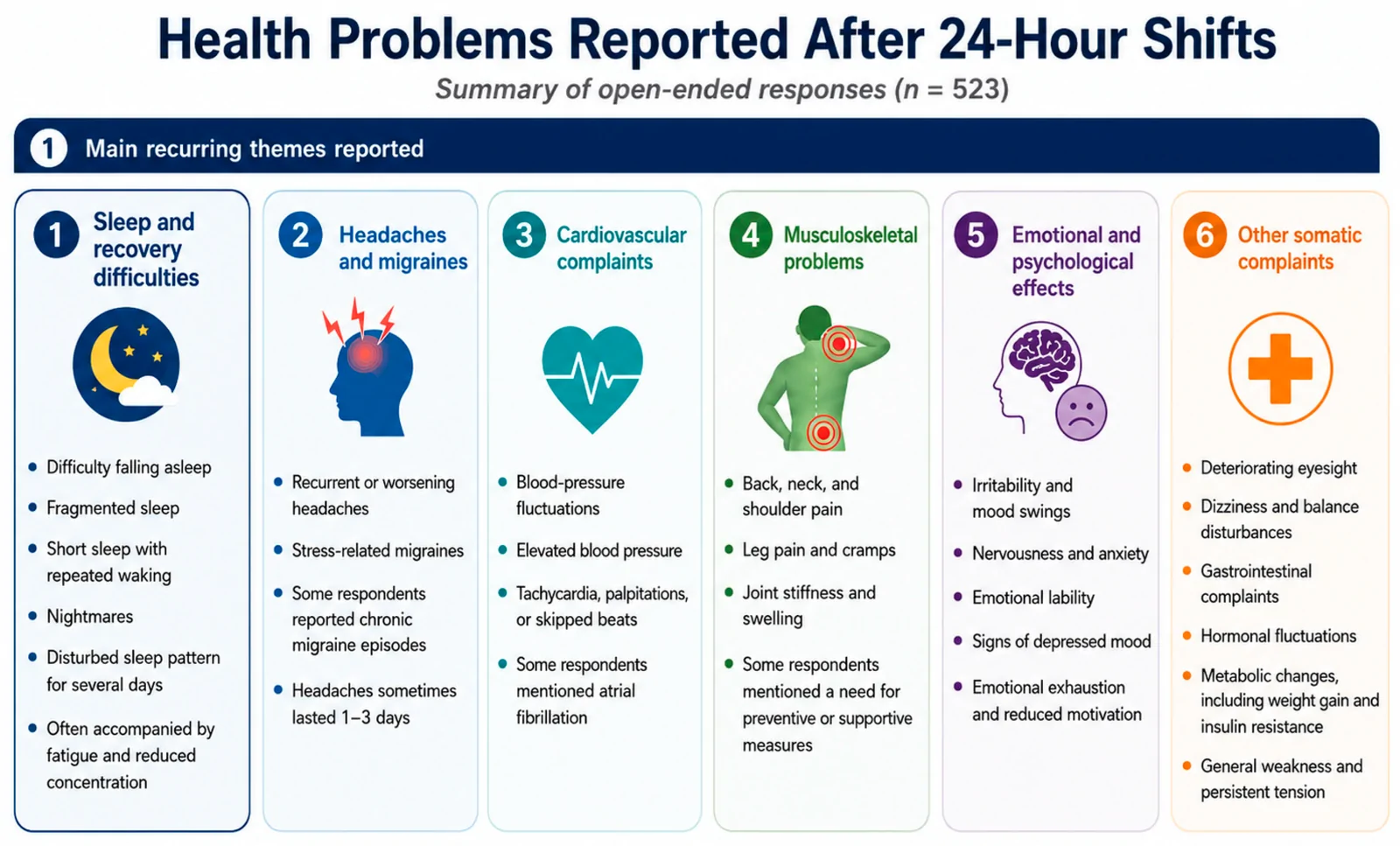
Health problems observed after 24-h shifts: summary of open-ended responses (n = 523).

In the first open-ended question, respondents were asked to describe health problems they had experienced or observed following 24-h shifts. A total of 523 responses were received. Inductive content analysis identified several recurring themes related to physical symptoms, fatigue, sleep and recovery difficulties, and emotional well-being. The main themes are presented in Figure 4.

#### 3.5.2. Reasons for Supporting or Opposing Revision of 24-h Shifts

**Figure 5:**
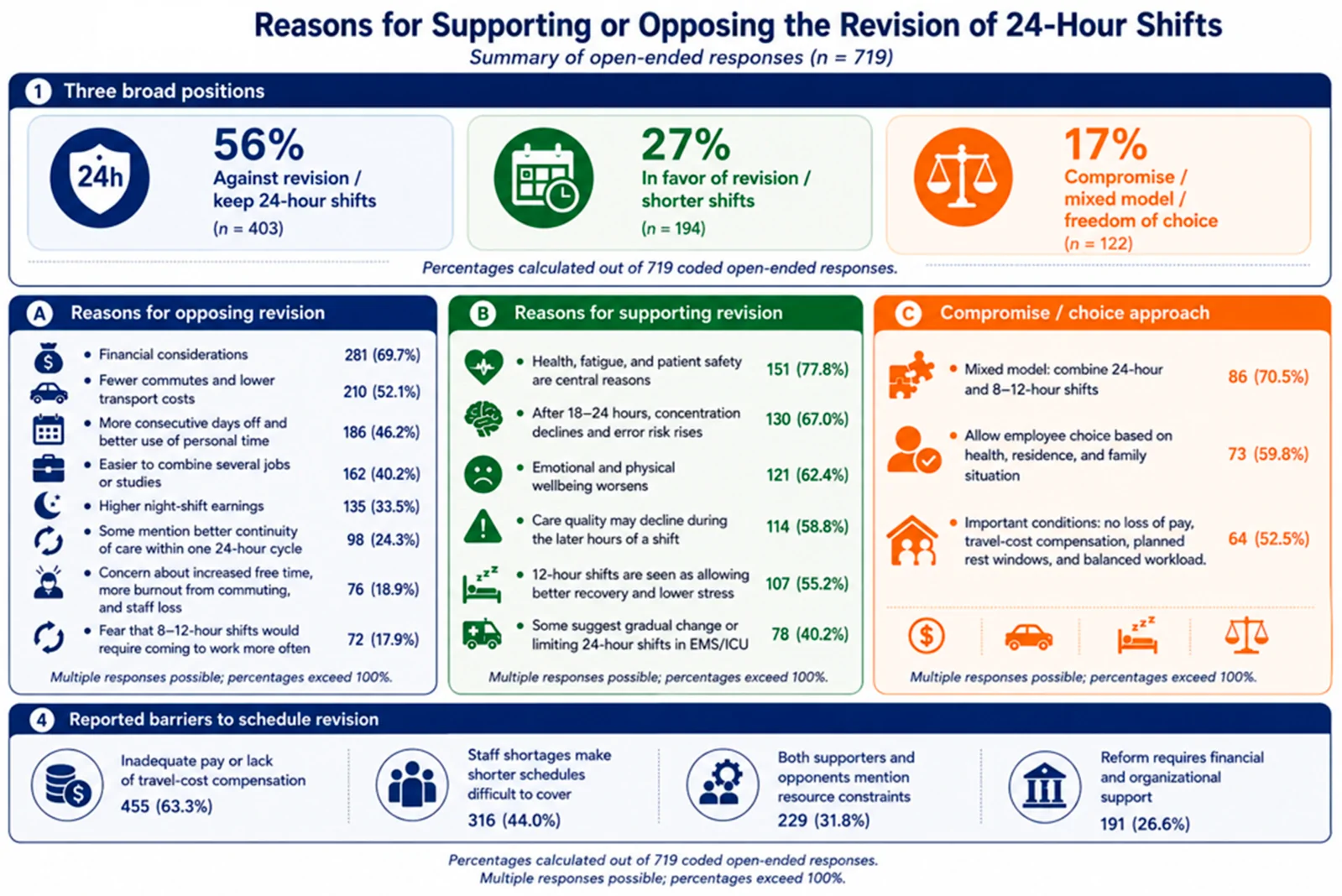
Supporting and opposing reasons.

The question “Why do you support or oppose revising 24-h shifts?” received 719 open-ended responses. Inductive content analysis identified three broad positions: opposition to revision, reflecting a preference to retain 24-h shifts; support for revision and transition to shorter shifts; and a conditional or compromise position favouring a mixed scheduling model or employee choice. Overall, 403 responses (56.1%) reflected opposition to revision, 194 (27.0%) supported revision, and 122 (17.0%) favoured a mixed or choice-based approach (Figure 5). Percentages for the specific reasons presented within each of these three categories were calculated using the respective category total as the denominator.

Percentages for the three broad positions were calculated using all 719 coded responses as the denominator. Percentages for specific reasons within each position were calculated using the corresponding category total (opposition: n = 403; support: n = 194; compromise/choice: n = 122). Percentages for reported barriers were calculated using all 719 responses. Multiple responses were possible; therefore, percentages within sections may exceed 100%.

#### 3.5.3. Anticipated Impact on Daily Work and Patient Care

For the open-ended question concerning the anticipated impact of revising 24-h shifts on daily work and patient care, 811 responses were received. Of these, 325 responses (40.1%) described potential benefits, while 486 responses (59.9%) emphasized potential negative consequences or practical concerns.

Reported potential benefits included better rest and recovery, reduced fatigue and perceived error risk, improved concentration, empathy, and communication with patients, and lower end-of-shift exhaustion. In contrast, frequently reported concerns included more frequent commuting and higher transport costs, fewer consecutive days off, reduced time for family life, greater difficulty combining several workplaces or studies, more frequent handovers, and concerns regarding staffing shortages and work-schedule organization.

#### 3.5.4. Suggestions for Reducing Fatigue from 24-h Shifts

The open-ended question concerning suggestions for reducing fatigue associated with 24-h shifts received 636 responses. The most frequently reported theme was adequate staffing and fair workload, mentioned in 543 responses (85.4%), followed by competitive pay and financial support in 437 responses (68.7%) and protected rest during shifts in 406 responses (63.8%). Work-schedule management was mentioned in 210 responses (33.0%), health and well-being support in 117 responses (18.4%), and better organization and practical support in 110 responses (17.3%). As individual responses could include suggestions relating to more than one theme, the percentages do not sum to 100%.

#### 3.5.5. Suggestions for Improving the Work Schedule and Additional Comments

The open-ended question concerning suggestions for improving work-schedule organization received 583 responses. The most frequently identified theme was a flexible mixed shift model, mentioned in 321 responses (55.1%), followed by a fair distribution of shifts and workload in 218 responses (37.4%). Protected rest and recovery were mentioned in 115 responses (19.7%), predictable but adaptable scheduling in 96 responses (16.5%), adequate staffing and support roles in 91 responses (15.6%), and pay and supportive management in 57 responses (9.8%). As individual responses could include more than one suggestion, the percentages do not sum to 100%.

The final open-ended question invited respondents to provide additional comments regarding 24-h shift work and related working conditions, and 541 responses were received. Pay and workload were the most frequently identified theme, appearing in 507 responses (93.7%). Staff sufficiency was mentioned in 217 responses (40.1%), followed by management attitudes and communication in 189 responses (34.9%), support measures in 141 responses (26.1%), differing perceptions of 24-h shift work in 67 responses (12.4%), and expressions of gratitude or appreciation in 59 responses (10.9%). As individual responses could contain more than one theme, the percentages do not sum to 100%.

## 4. Discussion

This study provides insight into Latvian healthcare workers’ experiences and attitudes toward 24-h shifts. The main finding is a clear contradiction between the perceived health and safety consequences of prolonged working hours and employees’ willingness to retain this work pattern. Although respondents working extended shifts frequently reported substantial fatigue, impaired well-being, and prolonged recovery, more than half opposed replacing 24-h shifts with shorter schedules. These findings suggest that attitudes toward shift reform reflect not only occupational health considerations but also financial, organizational, and personal considerations.

The reported burden associated with extended shift work was considerable. More than half of respondents (54.9%) had noticed health changes that they perceived as potentially associated with extended shift work, while 25.9% had consulted a physician for health issues they perceived as possibly related to this work schedule. These findings are consistent with recent evidence showing associations between extended working hours, insufficient recovery, occupational fatigue, sleep disturbance, physical health problems, and reduced cognitive performance among nurses [14,15]. A recent critical review also concluded that even 12-h shifts may be associated with increased fatigue and less favourable outcomes related to nurses’ physical health, burnout, job satisfaction, and intention to leave [16].

The findings also indicate a considerable emotional burden. More than half of respondents agreed that extended shifts negatively affected their emotional well-being, 61.1% reported emotional exhaustion, and 49.2% experienced persistent fatigue during the previous six months. These results support previous Latvian findings showing relationships between nursing workload, moral distress, burnout, and turnover intentions [4]. However, the present study did not assess burnout using a validated instrument; therefore, emotional exhaustion and persistent fatigue should be interpreted as self-reported experiences rather than as evidence of burnout. They are also consistent with a recent systematic review and meta-analysis involving 288,581 nurses, which found that nurse burnout was associated with poorer patient safety, more adverse events and medication errors, lower quality of care, and lower patient satisfaction [17].

Patient-safety findings were less uniform but remain important to consider. Approximately one-third of respondents reported that fatigue had contributed to an error or a perceived decline in care quality. Furthermore, 37.4% indicated that after more than 20 h of work they sometimes felt unable to make correct professional decisions. At the same time, more than half did not perceive a substantial effect of fatigue on their decision-making ability. This discrepancy may reflect adaptation to prolonged working hours or normalization of fatigue-related risk in settings where 24-h shifts are routinely used; however, this interpretation cannot be confirmed from the present cross-sectional data. Subjective confidence also does not necessarily exclude impaired attention or reduced cognitive performance. A recent scoping review found that fatigue was identified as a contributing factor in medication administration errors or near misses in 82% of the included studies [1].

The open-ended responses help explain this apparent contradiction. Respondents emphasized fewer commutes, longer periods of consecutive free time, higher earnings, and the opportunity to combine several workplaces. This is particularly relevant because 40.5% of respondents worked at more than one institution, and approximately 15% reported an overall workload exceeding 1.6 full-time equivalents. These findings suggest that a transition to shorter shifts without corresponding financial and organizational adjustments may be perceived as increasing commuting demands, reducing income, complicating family life, and potentially affecting employees’ willingness to remain at their current workplace. Previous research similarly indicates that nurses’ shift preferences are associated with personal circumstances, work–life balance, perceived flexibility, and opportunities to participate in scheduling decisions [18,19].

The findings regarding professional retention are also important. More than one-third of respondents had considered leaving the profession during the previous year. Inadequate pay was the most frequently identified demotivating factor, followed by excessive workload, chronic stress, and limited career-development opportunities. This suggests that shift duration should not be addressed as an isolated problem. Evidence indicates that staffing, workload, overtime, organizational support, and working conditions are associated with nurses’ job satisfaction and intention to leave [20,21]. Adequate registered-nurse staffing has also been associated with better patient outcomes and lower risk of adverse outcomes [22].

These findings have several implications for policy and hospital management. The results suggest that any consideration of revising 24-h shift practices should take into account remuneration, staffing capacity, work organization, and employee preferences. A gradual and differentiated approach may warrant further evaluation rather than an immediate universal replacement of 24-h shifts. Potential approaches could include evaluating mixed 8-, 12-, 16-, and 24-h scheduling models; considering shorter shifts in high-workload settings such as intensive care and emergency departments; reviewing the frequency of consecutive long shifts; ensuring opportunities for protected rest; and monitoring fatigue, self-reported safety concerns, absenteeism, and staff turnover. Employees may also benefit from involvement in the development of new schedules, as participation and schedule autonomy have been associated with greater acceptance of organizational changes [14,16,17].

Financial considerations emerged as an important issue in respondents’ preferences regarding potential schedule reform. Shorter shifts that result in substantial income reduction, increased transport expenses, or reduced recovery time may be less acceptable to employees. Salary reform, night-work compensation, travel support for employees living outside major cities, and adequate staffing may therefore need to be considered alongside changes in shift duration. Without such measures, schedule reform could potentially have unintended consequences for staffing and workforce retention.

This study has several strengths, including its large sample, the inclusion of different healthcare professions and clinical departments, and the combination of quantitative and open-ended responses. Nevertheless, the findings should be interpreted in light of several limitations. Participation was voluntary, and the sample cannot be considered fully representative of all Latvian healthcare workers. Nurses and women constituted the majority of respondents, potentially limiting the generalizability of the findings to other professional groups. The cross-sectional design does not allow causal relationships to be established, and health problems, fatigue, errors, and attitudes were self-reported rather than objectively measured. In addition, the study used a questionnaire developed specifically for this research rather than a fully validated standardized instrument, which may limit comparability with other studies. The qualitative analysis of open-ended responses may also be influenced by respondents’ subjective interpretations and researcher judgement, although responses were reviewed and grouped by several healthcare professionals. Because the analyses were descriptive, independent associations between participant characteristics and preferences regarding 24-h shifts were not examined.

Overall, the findings reveal a complex balance between respondents’ perceived occupational health and patient-safety concerns and the practical advantages associated with 24-h shifts. Respondents working extended shifts frequently reported fatigue, emotional exhaustion, health complaints, and perceived concerns regarding care quality. However, many participants continued to prefer 24-h shifts because of financial benefits, fewer commutes, longer periods of free time, and the possibility of combining several workplaces. These findings suggest that future consideration of schedule reform should take into account not only shift duration but also flexibility, staffing capacity, financial implications, recovery opportunities, and employee preferences.

## 5. Conclusions

This study identified a clear tension between healthcare workers’ perceived health and patient-safety concerns associated with extended shift work and the practical and financial advantages associated with retaining 24-h shifts. Respondents working extended shifts frequently reported fatigue, emotional exhaustion, health complaints, prolonged recovery, and perceived concerns regarding care quality and professional decision-making. At the same time, a substantial proportion preferred to retain 24-h shifts, particularly because of fewer commutes, longer periods of consecutive time off, financial considerations, and the possibility of combining employment across multiple workplaces.

The findings suggest that potential changes to 24-h shift practices should be considered within the broader context of staffing capacity, remuneration, workload, recovery opportunities, work–life balance, and employee preferences. Rather than supporting a single scheduling solution, the results highlight the importance of further evaluating flexible and mixed scheduling approaches and involving healthcare professionals in the development of future work-schedule models. Prospective studies are needed to determine the effects of alternative scheduling models on employee health, patient safety, and workforce retention.

## Figures and Tables

**Table 1:** Frequency of symptoms reported after extended shifts (16–24 h).

Symptom	Never	Rarely	Often	Hard to Say
Vision or coordination disturbances	421 (31.9%)	776 (58.9%)	103 (7.8%)	18 (1.4%)
Headaches	649 (49.2%)	353 (26.8%)	307 (23.3%)	9 (0.7%)
Blood-pressure fluctuations	480 (36.4%)	671 (50.9%)	156 (11.8%)	11 (0.8%)
Body aches or stiffness	554 (42.0%)	356 (27.0%)	404 (30.7%)	4 (0.3%)

**Table 2:** Perceived effects of long working hours on emotional and professional functioning.

Aspect	No Effect	Slight Effect	Moderate Effect	Substantial Effect	Very Substantial/Very Negative Effect	Hard to Say
Emotional stability	284 (21.5%)	350 (26.6%)	229 (17.4%)	279 (21.2%)	170 (12.9%)	6 (0.5%)
Ability to be empathetic toward patients	521 (39.5%)	295 (22.4%)	228 (17.3%)	189 (14.3%)	80 (6.1%)	5 (0.4%)
Mood during work	394 (29.9%)	329 (25%)	262 (19.9%)	242 (18.4%)	84 (6.4%)	7 (0.5%)
General stress level	295 (22.4%)	348 (26.4%)	220 (16.7%)	281 (21.3%)	169 (12.8%)	5 (0.4%)
Motivation to continue working in the profession	550 (41.7%)	226 (17.1%)	190 (14.4%)	206 (15.6%)	141 (10.7%)	5 (0.4%)

**Table 3:** Respondents’ ratings of factors considered when choosing to work 24-h shifts.

Factor	No Effect	Slight Effect	Moderate Effect	Substantial Effect	Very Strong Effect	Hard to Say
Financial consideration	133 (10.1%)	132 (10%)	186 (14.1%)	719 (54.6%)	132 (10%)	16 (1.2%)
Schedule convenience/days off after a shift	114 (8.6%)	100 (7.6%)	192 (14.6%)	783 (59.4%)	115 (8.7%)	14 (1.1%)
Pressure from colleagues or management	781 (59.3%)	171 (13.0%)	151 (11.5%)	130 (9.9%)	53 (4%)	32 (2.4%)
Feeling that there are no other real options	592 (44.9%)	174 (13.2%)	188 (14.3%)	240 (18.2%)	96 (7.3%)	28 (2.1%)
Internal motivation and professional loyalty	456 (34.6%)	228 (17.3%)	304 (23.1%)	253 (19.2%)	48 (3.6%)	29 (2.2%)

## Data Availability

The data are not publicly available and may only be provided by the corresponding author upon reasonable request, subject to applicable confidentiality, ethical, and data protection requirements.

## Supplementary Materials

The following supporting information can be downloaded at https://www.mdpi.com/article/10.3390/healthcare14182993/s1.

## Abbreviations

The following abbreviations are used in this manuscript:

IBM SPSS	Statistical Package for the Social Sciences
PĒK	Pētījuma Ētikas komitēja
n	Number of Participants
fig.	Figure
ICU	Intensive Care Unit
EMS	Emergency Medical Services
FTE	Full-Time Equivalent
