# Supplementary material for: Healthcare Workers’ Perspectives on 24-h Shift Work in Latvia: A Cross-Sectional Study
Source: Healthcare (Basel). 2026 Sep 13;14(18):2993. doi: 10.3390/healthcare14182993 (PMC13606737; doi:10.3390/healthcare14182993)
Supplement: Supplementary file 1 [file healthcare-14-02993-s001.zip › healthcare-4502934-supplementary.pdf]

Veselības aprūpes darbinieku (aprūpes un aprūpes atbalsta) pieredze un viedoklis par 24 stundu maiņu darbu slimnīcās

Cienījamie kolēģi!

Aicinām Jūs piedalīties aptaujā par 24 stundu darba maiņu praksi Latvijā, tās ietekmi uz veselību, profesionālo labsajūtu, pacientu aprūpes kvalitāti un darba organizāciju ārstniecības iestādēs.

Šī aptauja tiek īstenota **sadarbībā ar Latvijas Māsu asociāciju un Veselības ministrijas Cilvēkresursu nozares attīstības departamentu**. Aptaujas mērķis ir **izzināt māsu pieredzi un viedokli**, lai labāk izprastu, kādas ir 24 stundu darba maiņu priekšrocības un izaicinājumi.

Svarīgi uzsvērt, ka šī aptauja **nav lēmums par izmaiņām** un nenozīmē, ka tuvākajā laikā tiks pieņemti konkrēti lēmumi par 24 stundu maiņu atcelšanu vai saglabāšanu. Tās vienīgais mērķis ir **noskaidrot māsu viedokli** un izvērtēt iespējamos scenārijus nākotnes darba organizācijas uzlabojumiem.

Aptauja ir anonīma – Jūsu atbildes tiks apkopotas tikai kopsavilkuma veidā un izmantotas pētniecības, darba organizācijas pilnveides un stratēģiskās plānošanas vajadzībām. Jūs varat jebkurā brīdī atteikties no dalības. Aptaujas aizpildīšana aizņems aptuveni 15–20 minūtes.

Lūdzam atbildēt godīgi – Jūsu viedoklis ir ārkārtīgi nozīmīgs!

Pateicamies par Jūsu ieguldījumu, palīdzot veidot ilgtspējīgu, cilvēkcentrētu un drošu veselības aprūpes sistēmu Latvijā!

*Norāda nepieciešamo jautājumu

1. Jūsu vecums (lūdzu atzīmēt pilnus gadus): *

2. Jūsu dzimums: *

Atzīmējiet tikai vienu variantu.

- Sieviete
- Vīrietis
- Nevēlos norādīt
- Citas: ___

3. Jūsu izglītība: *

Atzīmējiet tikai vienu variantu.

- Vidējā profesionālā izglītība
- Pirmā līmeņa profesionālā augstākā izglītība
- Otrā līmeņa profesionālā augstākā izglītība vai bakalaura grāds
- Maģistra grāds (māszinības, pedagoģija, psiholoģija, vadība u.t.t.)
- Doktora grāds (medicīna un/vai citā nozarē)
- Citas: ___

4. Jūsu darba stāžs veselības aprūpē (lūdzu atzīmēt pilnus gadus): *

____

5. Jūsu amats: *

Atzīmējiet tikai vienu variantu.

- Jaunākais māsu palīgs
- Māsu palīgs
- Medicīnas asistents
- Radiologa asistents
- Ārstu palīgs
- Radiografers
- Vecmāte
- Māsa
- Atbildīgā māsa
- Virsmāsa
- Citas: ____

6. Pašreizējā darba struktūrvienība (piem., Ķirurģijas nodaļa, Intensīvā terapija u.c.): *

7. Jūsu darba slodze Slimnīcā *

Atzīmējiet tikai vienu variantu.

- Līdz 0,25 slodze
- 0,26 - 0,5 slodze
- 0,55 - 0,75 slodze
- 0,76 - 1,0 slodze
- 1,1 - 1,25 slodze
- 1,26 - 1,5 slodze
- 1,6 slodze un vairāk
- Akordadarbs
- Citas:

8. Jūsu **kopējā** darba slodze veselības aprūpē (tsk. pie vairākiem darba devējiem, izglītības process, piemēram pasniedzējs, docētājs kādā no izglītības iestādēm) *

Atzīmējiet tikai vienu variantu.

- Līdz 0,25 slodze
- 0,26 - 0,5 slodze
- 0,55 - 0,75 slodze
- 0,76 - 1,0 slodze
- 1,1 - 1,25 slodze
- 1,26 - 1,5 slodze
- 1,6 slodze un vairāk
- Akordadarbs
- Citas:

9. Jūs strādājat (kopējais darba vietu (vairākas iestādes) skaits veselības aprūpē): *

Atzīmējiet tikai vienu variantu.

- 1
- 2
- 3
- 4
- Citas:

10. Jūs strādājat papildus darbu izglītības iestādē, kā pasniedzējs, docētājs, lektors un vai citā amatā: *

Atzīmējiet tikai vienu variantu.

- Jā
- Nē
- Citas:

11. Kāds ir Jūsu darba grafiks Slimnīcā: *

Atzīmējiet tikai vienu variantu.

- Dienas darbs (7/8/10/12 stundas dienā)
- Darbs maiņas/dežūras (16 vai 24 stundas)
- Gan ikdienas darbs, gan darba maiņas
- Tikai nakts darbs

12. Darba slodzes izjūta pēdējā mēneša laikā: *

Atzīmējiet tikai vienu variantu.

- Ļoti zems
- Zems
- Vidēja
- Augsta
- Ļoti augsta

Pašreizējā pieredze ar 24 stundu maiņu darbu:

13. Cik bieži Jums ir 24 stundu maiņu darbs? *

Atzīmējiet tikai vienu variantu.

- Vairākas reizes nedēļā
- Apmēram reizi nedēļā
- Dažas reizes mēnesī (1–3 reizes)
- Retāk nekā reizi mēnesī
- Nekad

14. Cik bieži Jums ir 8 un/vai 12 stundu maiņu darbs? *

Atzīmējiet tikai vienu variantu.

- Vairākas reizes nedēļā
- Apmēram reizi nedēļā
- Dažas reizes mēnesī (1–3 reizes)
- Retāk nekā reizi mēnesī
- Nekad

15. Cik bieži Jums ir 16 stundu maiņu darbs? *

Atzīmējiet tikai vienu variantu.

- Vairākas reizes nedēļā
- Apmēram reizi nedēļā
- Dažas reizes mēnesī (1–3 reizes)
- Retāk nekā reizi mēnesī
- Nekad

16. Vai 24 stundu maiņu darba laikā Jums parasti ir iespēja īslaicīgi atpūsties vai pagulēt? *

Atzīmējiet tikai vienu variantu.

- Jā, gandrīz vienmēr (ir iespēja atvēlēt laiku miegam/atpūtai)
- Daļēji (reizēm izdodas nedaudz atpūsties)
- Bieži
- Reti
- Nē, parasti nav iespējas atpūsties

17. Vai pēc 24 stundu maiņu darba Jūs plānojat/ieplānojat nākamo maiņas darbu? *

Atzīmējiet tikai vienu variantu.

- Jā, gandrīz vienmēr
- Daļēji
- Bieži
- Reti
- Nē

18. Vai Jūs jūtat pietiekamu atbalstu no kolēģiem un/vai vadības, strādājot 24st. maiņas darbu? *

Atzīmējiet tikai vienu variantu.

- Jā, atbalsts ir pietiekams
- Daļēji – atbalsts ir neliels vai neregulārs
- Nē, atbalsts pārsvarā netiek nodrošināts

19. Cik grūti Jums personīgi ir strādāt 24st. maiņas darbu? *

Atzīmējiet tikai vienu variantu.

- Pavisam viegli
- Diezgan viegli
- Vidēji grūti
- Grūti
- Ļoti grūti

Ietekme uz veselību un labsajūtu:

20. Kā Jūs raksturotu savu pašsajūtu uzreiz pēc 24st maiņas darba? *

Atzīmējiet tikai vienu variantu.

- Jūtos labi atpūties, bez būtiska noguruma
- Jūtos nedaudz noguris, bet varu turpināt ierastās aktivitātes
- Jūtos ļoti noguris, nepieciešama ilgāka atpūta
- Jūtos pilnīgi izsmelts/pārguris, atgūšanās prasa ļoti ilgu laiku

21. Atzīmējiet, cik lielā mērā piekrītat apgalvojumam: " 24st. maiņas darba negatīvi ietekmē manu fizisko veselību." *

Atzīmējiet tikai vienu variantu.

- Pilnīgi nepiekrītu
- Drīzāk nepiekrītu
- Grūti pateikt
- Drīzāk piekrītu
- Pilnīgi piekrītu

22. Novērtējiet savu noguruma līmeni pēc 24st. maiņas darba skalā no 1 līdz 5 (1 – nejūtu nogurumu; 5 – esmu pilnībā izsmelts): *

Atzīmējiet tikai vienu variantu.

	1	2	3	4	5	
Nejū						Esmu pilnībā izsmelts

23. Cik bieži Jums pēc garas (24 stundu) maiņas darba rodas fiziskas sūdzības, piemēram, galvassāpes vai asinsspiediena paaugstināšanās? *

Atzīmējiet tikai vienu variantu.

- Nekad
- Reti
- Dažreiz
- Bieži
- Ļoti bieži

24. Atzīmējiet, cik lielā mērā piekrītat apgalvojumam: " 24st. maiņas darbs negatīvi ietekmē Jūsu emocionālo labsajūtu." *

Atzīmējiet tikai vienu variantu.

- Pilnīgi nepiekrītu
- Drīzāk nepiekrītu
- Grūti pateikt
- Drīzāk piekrītu
- Pilnīgi piekrītu

25. Novērtējiet, cik lielā mērā 24 stundu maiņas darbs ietekmē Jūsu emocionālo pašsajūtu (piemēram, stresa līmeni, garastāvokli, izdeguma sajūtu) (1 – neietekmē nemaz, 5 – ietekmē ļoti negatīvi): *

Atzīmējiet tikai vienu variantu.

	1	2	3	4	5	
Neie						Ietekmē ļoti negatīvi

26. Vai Jūs esat novērojis/-usi izmaiņas savā veselības stāvoklī, ko varētu saistīt ar 24st. maiņas darbu? *

Atzīmējiet tikai vienu variantu.

- Jā
- Nē

27. Ja iepriekšējā jautājumā Jūs atbildējāt ***Jā***, lūdzu īsi aprakstiet, kādas problēmas esat pamanījis/-usi (piemēram, miega traucējumi, biežākas galvassāpes, asinsspiediena svārstības u.c.): *

28. Vai noguruma dēļ 24st. maiņa darbā Jums jebkad ir gadījies pieļaut kļūdu darbā vai just, ka aprūpes kvalitāte pasliktinās? *

Atzīmējiet tikai vienu variantu.

- Jā, ir gadījies
- Nē, nekad
- Grūti pateikt

29. Cik lielā mērā, pēc Jūsu domām, nogurums 24 stundu maiņas darbā palielina risku pieļaut kļūdas darbā vai apdraudēt pacientu drošību? (1 – nepalielina nemaz, 5 – palielina ļoti būtiski): *

Atzīmējiet tikai vienu variantu.

- Nepalielina nemaz
- Palielina nedaudz
- Palielina merēni
- Palielina būtiski
- Palielina ļoti būtiski

30. Cik ilgs atpūtas laiks Jums parasti nepieciešams, lai pilnībā atgūtos pēc 24st. maiņas darba? *

Atzīmējiet tikai vienu variantu.

- Mazāk nekā 12 stundas
- Apmēram 12–24 stundas
- Apmēram 1 pilna diena (līdz nākamās dienas vakaram)
- Apmēram 2 dienas
- Vairāk nekā 2 dienas

31. Novērtējiet Jūsu miega kvalitāti pirmajā miegā pēc 24st. maiņas darba (1 – ļoti slikta, 5 – ļoti laba): *

Atzīmējiet tikai vienu variantu.

	1	2	3	4	5	
Ļoti						Ļoti laba

32. Cik viegli Jums ir saskaņot 24 stundu maiņas darba grafiku ar ģimenes dzīvi un personīgajām vajadzībām?: *

Atzīmējiet tikai vienu variantu.

- Ļoti grūti
- Grūti
- Neitrāli
- Diezgan viegli
- Viegli

33. Novērtējiet, kā maiņas darbs ietekmē Jūsu profesionālā gandarījuma un darba jēgpilnuma sajūtu (1 – ļoti mazinās jēgpilnuma sajūta, 5 – neietekmē, saglabājas tādā pašā līmenī kā parasti): *

Atzīmējiet tikai vienu variantu.

	1	2	3	4	5	
Ļoti	⬭	⬭	⬭	⬭	⬭	Neietekmē, saglabājas tādā pašā līmenī kā parasti

34. Cik ilgi Jūs parasti guļat pirmajā naktī pēc 24 stundu maiņas darba? *

Atzīmējiet tikai vienu variantu.

- ⬭ Mazāk par 3 stundām
- ⬭ 3–5 stundas
- ⬭ 6–7 stundas
- ⬭ 8 vai vairāk stundas
- ⬭ Citas: ____

35. Cik bieži pēc 24st. maiņas darba Jums ir grūtības aizmigt vai miegs ir traucēts? *

Atzīmējiet tikai vienu variantu.

- ⬭ Nekad
- ⬭ Reti
- ⬭ Reizēm
- ⬭ Bieži
- ⬭ Vienmēr

36. Cik bieži Jūs izjūtat šādus simptomus pēc 24st. maiņas darba? (atzīmēt biežumu katram): *

Katrā rindiņā atzīmējiet tikai vienu variantu.

	Nekad	Reizēm	Bieži
**Redzes vai koordinācijas traucējumi**	◯	◯	◯
**Galvassāpes**	◯	◯	◯
**Asinsspiediena svārstības**	◯	◯	◯
**Ķermeņa sāpes vai stīvums:**	◯	◯	◯

37. Vai Jūs kādreiz esat apmeklējis ārstu veselības traucējumu dēļ, kurus, iespējams, izraisīja 24st. maiņu darba grafiks?: *

Atzīmējiet tikai vienu variantu.

◯ Jā

◯ Nē

38. Novērtējiet, kā garās maiņu darba stundas ietekmē šādus aspektus (1 – neietekmē, 5 – ļoti negatīvi ietekmē): *

Katrā rindiņā atzīmējiet tikai vienu variantu.

	Neietekmē	Ietekmē nedaudz	Ietekmē mēreni	Ietekmē būtiski	Ļoti negatīvi ietekmē
**Emocionālo stabilitāti:**	⬭	⬭	⬭	⬭	⬭
**Spēju būt empātiskam pret pacientiem**	⬭	⬭	⬭	⬭	⬭
**Noskaņojumu darba laikā**	⬭	⬭	⬭	⬭	⬭
**Vispārēju stresa līmeni**	⬭	⬭	⬭	⬭	⬭
**Motivāciju turpināt darbu profesijā**	⬭	⬭	⬭	⬭	⬭

39. Lūdzu, norādiet, vai pēdējo 6 mēnešu laikā esat izjutuši kādu no turpmāk minētajām pazīmēm: *

Atzīmējiet visus atbilstošos variantus.

- ☐ Pastāvīgs nogurums
- ☐ Cinisms vai attālināšanās no pacientiem
- ☐ Sajūta, ka jūsu darbs nav jēgpilns
- ☐ Koncentrēšanās grūtības
- ☐ Emocionāla izsīkuma sajūta

40. Cik lielā mērā 24st. maiņu darbs izjauc Jūsu ģimenes vai sociālo dzīvi? *

Atzīmējiet tikai vienu variantu.

- Nemaz
- Nedaudz
- Mēreni
- Būtiski
- Ļoti būtiski

41. Vai Jūsu darba vietā ir noteikta kārtība, kā rīkoties gadījumos, kad darbinieks jūtas fiziski vai garīgi nespējīgs turpināt maiņa darbu? *

Atzīmējiet tikai vienu variantu.

- Jā
- Nē
- Nezinu

Attieksme pret iespējamu 24 stundu maiņu darba pārskatīšanu:

42. Vai Jūs atbalstāt 24 stundu maiņu darba pārskatīšanu Jūsu darba vietā, aizstājot tās ar īsākām darba stundām (piemēram, 8 vai 12 stundas)? *

Atzīmējiet tikai vienu variantu.

- Pilnībā atbalstu
- Drīzāk atbalstu
- Neitrāls (grūti pateikt)
- Drīzāk neatbalstu
- Pilnībā neatbalstu

43. Lūdzu, pamatojiet savu atbildi: kādēļ Jūs atbalstāt vai neatbalstāt 24st. maiņu darba pārskatīšanu?: *

44. Kā, Jūsuprāt, 24st. maiņu darba pārskatīšana varētu ietekmēt Jūsu ikdienas darbu un pacientu aprūpi? *

45. Vai Jūs atbalstāt 24 stundu maiņu darba pārskatīšanu visā valstī (Latvijā)? *

Atzīmējiet tikai vienu variantu.

- Jā, pilnībā atbalstu
- Drīzāk atbalstu
- Neitrāls / grūti pateikt
- Drīzāk neatbalstu
- Nē, esmu pret

46. Kāds būtu, Jūsuprāt, labākais maiņu darba stundu ilgums tiešajam darbam? *

Atzīmējiet tikai vienu variantu.

- Līdz 8 stundām
- 8–12 stundas
- Līdz 16 stundām
- 24 stundas
- Citas: ____

47. Kādi, Jūsuprāt, būtu ieguvumi, ja Latvijā pārskatītu 24 stundu maiņu darba? (atzīmējiet līdz 3 svarīgākajiem) *

Atzīmējiet visus atbilstošos variantus.

- [ ] Labāka darbinieku veselība un mazāks izdegšanas risks
- [ ] Lielāka pacientu drošība
- [ ] Uzlabots darba–dzīves līdzsvars
- [ ] Vienmērīgāka darba slodze
- [ ] Vairāk darba vietu (vairāk nepieciešamo maiņu)
- [ ] Nav būtisku ieguvumu
- [ ] Citas: ______________________

48. Kādi, Jūsuprāt, būtu riski vai problēmas, ja 24st. maiņu darbs tiktu pārskatīts? (atzīmējiet līdz 3 svarīgākajiem) *

Atzīmējiet visus atbilstošos variantus.

- [ ] Biežāk jāierodas darbā (vairāk maiņu nedēļā)
- [ ] Mazāks brīvais laiks starp maiņām
- [ ] Sarežģītāka darba grafika organizācija
- [ ] Samazināts atalgojums
- [ ] Iespējamais personāla trūkums
- [ ] Citas: ______________________

49. Vai Jūs uzskatāt, ka nogurums 24st. maiņu darba laikā ietekmē Jūsu spēju pieņemt drošus un precīzus lēmumus ārstniecībā? *

Atzīmējiet tikai vienu variantu.

- ( ) Jā, ievērojami
- ( ) Jā, nedaudz
- ( ) Nē, nejūtu būtisku ietekmi
- ( ) Grūti pateikt

50. Cik bieži pēc 20+ stundām darba Jūs jūtat, ka nespējat pieņemt korektus profesionālos lēmumus? *

Atzīmējiet tikai vienu variantu.

- Nekad
- Reizēm
- Bieži
- Vienmēr

51. Vai Jūs būtu vairāk motivēts(-a) strādāt īsākas darba maiņas, ja tās būtu finansiāli vienlīdzīgas ar 24st darba maiņu apmaksu? *

Atzīmējiet tikai vienu variantu.

- Jā
- Nē
- Varbūt

52. Kam Jūs dotu priekšroku, ja izvēle būtu Jūsu ziņā? *

Atzīmējiet tikai vienu variantu.

- Palikt pie 24st. maiņu darba ar kompensējošām brīvdienām
- Pāriet uz īsākām maiņu darba (8–12st.), pat ja nav papildu brīvdienu
- Jaukts modelis (daļēji 24st., daļēji īsākas darba maiņas)
- Nezinu

53. Kāds ir galvenais iemesls, kāpēc Jūs piekrītiet strādāt 24st. maiņu darba? (atzīmējiet līdz 3 galvenajiem) *

Atzīmējiet visus atbilstošos variantus.

- [ ] Lielāks atalgojums
- [ ] Ilgāks brīvais laiks pēc darba
- [ ] Trūkst personāla – lūdz strādāt tie;sais vadītājs
- [ ] Esmu pieradis pie šāda režīma
- [ ] Vadība nepieļauj īsākas darba maiņas
- [ ] Grafiks ļauj apvienot vairākus darbus
- [ ] Nav citas izvēles darba vietā
- [ ] Citas: ____________________

54. Vai Jūs šobrīd justos droši atteikties no 24st. maiņa darba, ja alternatīva būtu piedāvāta? *

Atzīmējiet tikai vienu variantu.

- ( ) Jā
- ( ) Nē
- ( ) Neesmu pārliecināts/-a

55. Novērtējiet, cik lielā mērā Jūsu izvēli strādāt 24st. maiņu darbs ietekmē šādi faktori (1 – neietekmē, 5 – ļoti ietekmē): *

Katrā rindiņā atzīmējiet tikai vienu variantu.

	Neietekmē	Ietekmē nedaudz	Ietekmē mēreni	Ietekmē būtiski	Ļoti negatīvi ietekmē
**Finansiālais apsvērums**					
**Grafika ērtība/brīvās dienas pēc dežūras**					
**Spiediens no kolēģiem vai vadības**					
**Sajūta, ka nav citu reālu variantu**					
**Iekšējā motivācija un profesionālā lojalitāte**					

Organizatoriskie aspekti un darba maiņu plānošana:

56. Cik lielā mērā Jūs varat ietekmēt savu darba grafiku (maiņu darba sadalījumu)? *

Atzīmējiet tikai vienu variantu.

- Pilnībā – varu izvēlēties
- Daļēji – varu ieteikt, bet nav garantijas
- Maz – reti tiek ņemti vērā mani priekšlikumi
- Vispār nē

57. Vai Jums ir bijusi iespēja paust savu viedokli par maiņu darba ilguma pārmaiņām Slimnīcas vai sv/nodaļas līmenī? *

Atzīmējiet tikai vienu variantu.

- Jā, mans viedoklis ir uzklausīts
- Daļēji – ir bijusi diskusija, bet bez rezultāta
- Nē, nekad nav jautāts
- Nezinu

58. Ko, Jūsuprāt, Slimnīca vai darba devējs varētu darīt, lai mazinātu 24st. maiņas darba radīto nogurumu vai sekas?:

_____

_____

_____

_____

_____

Ieteikumi Slimnīcai

59. Kādas pārmaiņas Jūs vēlētos ieviest Slimnīcas darba grafiku organizācijā, lai veicinātu Jūsu veselību un darba efektivitāti?:

____
____
____
____
____

Motivācija strādāt profesijā:

60. Kā Jūs šobrīd vērtējat savu motivāciju turpināt strādāt profesijā? *

Atzīmējiet tikai vienu variantu.

- Ļoti zema (nopietni apsveru atstāt profesiju)
- Drīzāk zema
- Vidēja
- Drīzāk augsta
- Ļoti augsta (esmu ļoti motivēts turpināt strādāt)

61. Vai pēdējā gada laikā esat apsvēris(-usi) domu pamest profesiju? *

Atzīmējiet tikai vienu variantu.

- Jā
- Nē
- Nezinu / grūti pateikt

62. Kādi faktori Jūs visvairāk motivē turpināt strādāt profesijā? (atzīmējiet visas atbilstošās atbildes) *

Atzīmējiet visus atbilstošos variantus.

- [ ] Aicinājums palīdzēt cilvēkiem (darbs ar pacientiem sniedz gandarījumu)
- [ ] Gandarījums par paveikto (profesionālais lepnums par padarīto darbu)
- [ ] Labi kolēģi un savstarpējs atbalsts kolektīvā
- [ ] Relatīvi elastīgs grafiks un brīvdienu iespējas (piem., vairāk brīvdienu pēc garas dežūras)
- [ ] Stabils, drošs darbs (pastāvīga darbavieta un ienākumi)
- [ ] Profesionālās izaugsmes un tālākizglītības iespējas
- [ ] Finansiālais atalgojums
- [ ] Citas: ______________________

63. Kurš faktors visvairāk mazina Jūsu motivāciju vai rada vēlmi pamest profesiju? *

Atzīmējiet visus atbilstošos variantus.

- [ ] Garās darba stundas un ar tām saistītais nogurums (t.sk. 24h maiņu darbs)
- [ ] Pārmērīga darba slodze un hronisks stress
- [ ] Nepietiekams atalgojums
- [ ] Novērtējuma trūkums darbavietā (no vadības vai pacientu puses)
- [ ] Ierobežotas karjeras un profesionālās izaugsmes iespējas
- [ ] Darba negatīvā ietekme uz veselību
- [ ] Citas: ______________________

Pateicamies, ka dalāties savā pieredzē un palīdzat veidot cilvēkcentrētu veselības aprūpes sistēmu!

64. Ja vēlaties, lūdzu, pievienojiet komentāru, lai dalītos ar papildu informāciju vai ierosinājumiem:

Uzņēmums Google nav šī satura autors un to neatbalsta.

Google Veidlapas
